# Salt Effect on the Antioxidant Activity of Red Microalgal Sulfated Polysaccharides in Soy-Bean Formula

**DOI:** 10.3390/md13106425

**Published:** 2015-10-20

**Authors:** Ariela Burg, Levy-Ontman Oshrat

**Affiliations:** Department of Chemical Engineering, Sami Shamoon College of Engineering, Basel/Bialik sts., Beer-Sheva 8410001, Israel

**Keywords:** antioxidants, calcium, microalgae, polysaccharides, *Porphyridium* sp., *Porphyridiumaerugineum*

## Abstract

Sulfated polysaccharides produced by microalgae, which are known to exhibit various biological activities, may potentially serve as natural antioxidant sources. To date, only a few studies have examined the antioxidant bioactivity of red microalgal polysaccharides. In this research, the effect of different salts on the antioxidant activities of two red microalgal sulfated polysaccharides derived from *Porphyridium* sp. and *Porphyridium aerugineum* were studied in a soy bean-based infant milk formula. Salt composition and concentration were both shown to affect the polysaccharides’ antioxidant activity. It can be postulated that the salt ions intefer with the polysaccharide chains’ interactions and alter their structure, leading to a new three-dimensional structure that better exposes antiooxidant sites in comparison to the polysaccharide without salt supplement. Among the cations that were studied, Ca^2+^ had the strongest enhancement effect on antioxidant activities of both polysaccharides. Understanding the effect of salts on polysaccharides’ stucture, in addition to furthering knowledge on polysaccharide bioactivities, may also shed light on the position of the antioxidant active sites.

## 1. Introduction

Antioxidants have a positive effect on human health as they can slow down oxidative stress processes caused by reactive oxygen species (ROS), e.g., OH, H_2_O_2_ and O_2_^−^ [1,2,3]. These ROS attack macromolecules such as membrane lipids, proteins and DNA [1,4], leading to many health disorders with severe tissue injuries such as cancer, diabetes mellitus, neurodegenerative and inflammatory diseases [5,6,7,8]. In the food industry, oxidation of lipids can lead to food product deterioration (e.g., in nutritional value, safety and appearance) due to formation of undesirable secondary lipid peroxidation products. Therefore, many synthetic commercial antioxidants such as butylated hydroxytoluene (BHT), butylated hydroxyanisole (BHA), tert-butylhydroquinone (TBHQ) and propyl gallate (PG) are used to retard the oxidation and peroxidation processes [9]. However, the use of these synthetic antioxidants must be under strict regulation due to potential health hazards [10,11,12], hence there is an ongoing search for safe, natural alternatives [13,14].

Recently, both the food and pharmaceutical industries have become interested in the development of antioxidants from natural sources. Sulfated polysaccharides derived from marine algae, which are known to exhibit many biological activities, may be a potential natural antioxidant source for these industries [15]. Among the scant studies conducted regarding the antioxidant potential of sulfated polysaccharides, most efforts were focused on those derived from seaweeds [16,17,18,19,20,21,22], whereas very little is known regarding red microalgal sulfated polysaccharides [23,24,25]. The mechanism of these sulfated polysaccharides’ antioxidant activity is not certain. It is also known that various residues which could bind to these polysaccharides, such as pigments, flavones, peptides, proteins, and polyphenols can improve their antioxidant activities [26].

Our research is focused on two soluble-sulfated polysaccharide fractions produced by red microalgal species—the seawater *Porphyridium* sp. (hereafter—PS1) and the freshwater *Porphyridium aerugineum* (hereafter—PS2)*.* These microalgae, in contrast to seaweeds, are encapsulated by a thick layer of mucilaginous sulfated polysaccharides that lack the rigid microfibrillar component typical in most of alga species [27]. During growth, the external fraction of these polysaccharides is released to the surrounding aqueous medium, where it accumulates and increases medium viscosity [28,29,30]. The precise structures of the soluble-sulfated polysaccharide fractions are not fully understood due to their complexity and the lack of known carbohydrolases that degrade them [30,31,32]. The soluble-sulfated polysaccharide fractions are of high molecular weight (~1–7 × 10^6^ g·mol^−1^), and due to the presence of glucuronic acid residues and sulfates they are anionic heteropolymers, containing approximately 10 different mono sugars, mainly glucose and galactose [33,34,35,36]. In addition, both are built from the same disaccharide building block [37]. PS1 was found to contain several proteins, which are non-covalently bound to it [38]. The most prominent protein detected was a 66-kDa glycoprotein; This protein consists of a polypeptide of approximately 58 kDa and glycan moieties of approximately 8 kDa [38,39]. Sequencing of a cDNA clone encoding the 66-kDa glycoprotein revealed that this is a novel protein, which lacks similarity to any protein in the public domain databases. However, in the SCOP databases, some structural similarities were found between the carbohydrate-binding domain (CBD) and protein superfamilies, such as glycosyltransferases, pectin lyase-like proteins, sialidases, and conA-like lectins/glucanases, indicating a possible role for this 66-kDa glycoprotein in synthesis/modification of the cell-wall polysaccharide [38]. In addition, the glycoprotein was shown to play a role in biorecognition [40].

Physicochemical analysis conducted on these soluble-sulfated polysaccharide fractions indicate that even at low concentrations they create highly viscous polymer solutions, compared to the viscosity of aqueous solutions containing polysaccharides such as xanthans or carrageenans [41]. It is believed that red microalgal polysaccharide layers protect the cells against drought, solar irradiation and maintain the humidity required by the cells [30].

PS1 is stable over a wide range of temperatures (30–160 °C), pH values (2–9), light and salinities [31,41,42,43,44,45,46,47,48,49]. Rheological studies have demonstrated that it is comprised of an oriented, single two-fold helical structure with a pitch of 1.6 nm, *i.e.*, a single chain helix with a regular chemical repeat [49]. The stiffness of PS1 was reported to be in the same rigid helical range as that of xanthan gum and DNA [43]. A conformational transition state in the PS1 chain was observed at low ionic strength (<0.01 M), most likely reflecting a contraction of the polymer chain from a highly stretched to a stiff, wormlike chain [43]. Furthermore, rheological studies have also indicated that the nature of PS1 is affected by ionic strength, valence, type and concentration of cations, pH, polymer concentration, and temperature [43]. For example, in a salt-free solution, PS1 favors a stretched chain conformation, the result of long-range electrostatic effects, but the addition of an electrolyte such as NaCl, masks the electrostatic interactions and allows a more flexible configuration resulting in lower intrinsic viscosities [43].

To our knowledge, only a few reports have evaluated the antioxidant activity of PS1 and PS2 [23,24]. These fractions were shown to exhibit dose-dependent antioxidant activity, and were found to serve as better inhibitors of linoleic acid auto-oxidation compared to carrageenan and cellulose [24]. In addition, PS1 inhibited the oxidative damage to 3T3 cells caused by FeSO_4_ in a dose-dependent manner [24]. A search for the active antioxidant component was also conducted; PS1 polysaccharide fragments created by microwave and sonication exerted an inhibitory effect on oxidative damage in dependence on the fraction received following fragmentation [23]. Thus, it seems that a significant part of the polysaccharide is not required for its antioxidant activity. The 66-kDa glycoprotein within PS1 may also play role in the polysaccharide’s antioxidant activity [24]. It was suggested that the antioxidant activity helps the cells cope with the ROS to which they are exposed under stress such as drought, extreme temperature, solar irradiation, *etc.* [24]. Taking together these findings, together with the fact that they are safe to use in food, are stable over a wide range of temperatures/environmental conditions, and have favorable rheological and chemical properties, the PS1 and PS2 polysaccharides may be used as antioxidant additives in food or/and biomedical products. Despite this potential of PS1 and PS2, knowledge on their antioxidant activity is still lacking, and the effect of salt on this activity has not been studied to date.

Herein, the effect of salt on the antioxidant activity of PS1 and PS2 in the presence of soy bean-based infant milk formula was studied. Infant milk formula based on soy bean was chosen as a good platform for oxidative stress because of its composition; it is rich in polyunsaturated fatty acids (PUFA) (Table S1 in supplementary information) which are sensitive to oxidative stress. The results of this study contribute to our understanding of red microalgal polysaccharide structure and function, especially as antioxidants, under various salt conditions.

## 2. Results and Discussion

**Figure 1:**
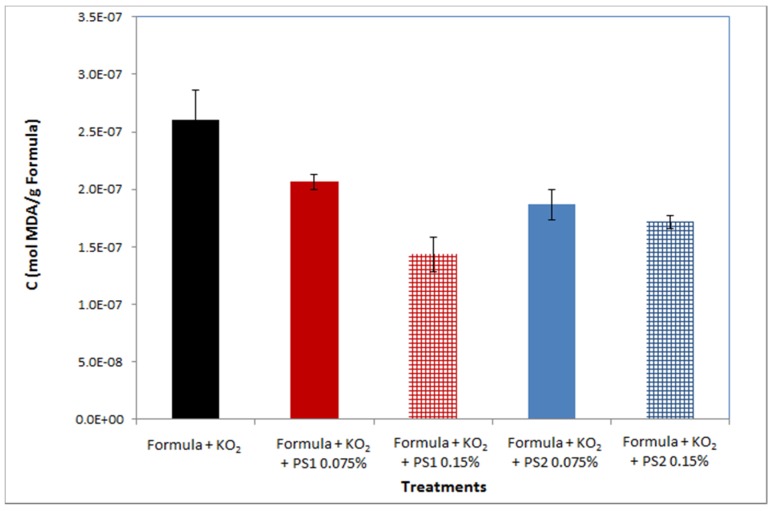
Antioxidant activity of PS1 and PS2 in soy bean milk formula following addition of KO_2_. C_MDA_ was measured by thiobarbitoric acid (TBA) method. All treatments contained 0.5 g formula, 0.35 mM KO_2_, with or without 0.075/0.15% *w*/*v* of either PS1 or PS2 polysaccharides. Values are expressed as mean ± SD, *n* = 3.

The antioxidant activity of red microalgal soluble-sulfated polysaccharide fractions in infant milk formula was evaluated by measuring the Malondialdehyde (MDA)-oxidant product after adding KO_2_ as an oxidative reagent. MDA levels were significantly reduced in oxidant-damaged formulas containing either of the polysaccharides, in comparison to the control, see Figure 1. MDA levels were also measured in pure PS solutions with and without KO_2_; in both cases MDA levels were negligible; thus it can be assumed that the polysaccharides are probably not affected by KO_2_—induced oxidant damage.

The effective antioxidant activity of polysaccharides derived from red microalgae that was demonstrated in infant milk formula (Figure 1) is in accordance with previous reports demonstrating the antioxidant activity of red microlagal polysaccharides [23,24,25]. The antioxidant activity of both soluble-sulfated polysaccharide fractions seems to be similar with a slight advantage to PS1. The increase in the polysaccharide concentration (PS1/PS2) also improved their antioxidant activity, but it seems that PS1 has a better dose-concentration effect than that of PS2 (Figure 1). The similarity in the antioxidant capability of these polysaccharides is not surprising since it is known that the structure and the physical behavior of the polysaccharides are similar; they are composed of the same building block with the same predominant monosaccharides and are both negatively charged. The relative advantage of PS1 may be attributed to differences between the polysaccharides, such as sulfate content, ratio of monosaccharides, *etc.* [41].

In order to expand our knowledge on the antioxidant activity of the polysaccharides, it is important to study their activities under different conditions. Ionic strength and cation type were found to influence the intrinsic viscosity of PS1, probably by varying the electrostatic forces and leading to conformational changes [43]. Herein, the effect of potassium, sodium and divalent cation salts with different anions on the antioxidant ability of polysaccharides was tested in an oxidant-damaged infant formula. Salts were added to the polysaccharides prior to their exposure to the oxidant-damaged infant formula, and MDA concentrations were measured as will be explained in the experimental section, to assess the effect of salts on the antioxidant activity of the polysaccharides. All results are displayed as MDA reduction percentages, relative to each experimental setup without the salt.

All types of salts tested, regardless of their anion/cation composition and their concentrations, positively induced the antioxidant activity of both polysaccharides in comparison to the native polysaccharide (control-without salt supplement). We were thus interested in elucidating the effect of different cations and anions, as well as different salt concentrations, on this induction.

**Figure 2:**
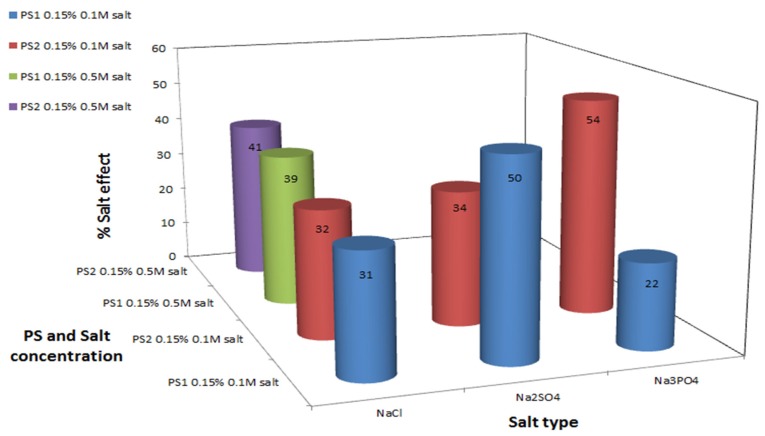
Polysaccharide antioxidant activity induction by sodium salts. C_MDA_ was measured by TBA method. Experimental setup included 0.5 g formula with 0.35 mM KO_2_, 0.15% *w*/*v* polysaccharide and various sodium salts at two different concentrations, in a final volume of 10 mL. Values represent the mean of at least 3 repeats; Maximum standard deviation equals 5%.

**Figure 3:**
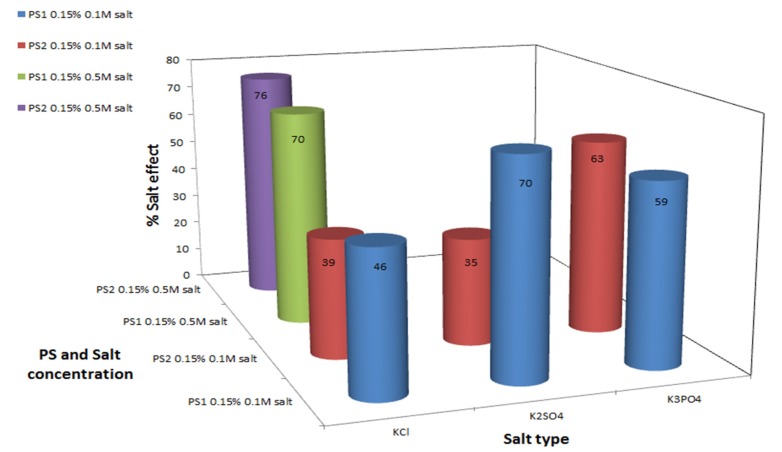
Polysaccharide antioxidant activity induction by potassium salts. C_MDA_ was measured by TBA method. Experimental setup included 0.5 g formula with 0.35 mM KO_2_, 0.15% *w*/*v* polysaccharide and various potassium salts at two different concentrations, in a final volume of 10 mL. Values represent the mean of at least 3 repeats; Maximum standard deviation equals 8%.

Monovalent cation type effect: In order to elucidate the effect of different monovalent cations on the polysaccharides’ antioxidant bioactivity, a comparison was performed between NaCl and KCl, between Na_2_SO_4_ and K_2_SO_4_ and between Na_3_PO_4_ and K_3_PO_4_. Generally, regardless of the anion present, the K^+^ cations were found to have a stronger influence on the antioxidant activity of the polysaccharides, inducing a marked improvement in MDA level reduction, in comparison to the Na^+^—formula treatments, as seen by comparing Figure 2 and Figure 3.

The cation antioxidant enhancement effect can be explained: cations derived from the salts bind to negatively charged residues in the polysaccharide, such as sulfate and glucuronic acid residues. This interaction with the polysaccharide, depending on the cation type, may change the polysaccharide’s three-dimensional structure due to conformational or structural changes in the solution, by masking the electrostatic repulsions between the polysaccharide chain molecules (which decreases association between the polysaccharide chains). The newly formed structure probably better exposes the potential antioxidant sites in comparison to a reduced-salt native polysaccharide. This postulation is based on a former viscometry study, which indicated that salts can change the polysaccharide conformational structure [43]. In our case, the potassium ions probably induced a change in the intermolecular bonds in the polysaccharide—leading to better exposure of the antioxidant sites in comparison to the three dimensional structure that was formed in the presence of sodium salts. It was expected that an incease in cation concentration will lead to stronger reduction of MDA levels. However, this is not always the case, as seen by comparing the results obtained for Na_2_SO_4_ with those obtained for Na_3_PO_4_ (Figure 2), and K_2_SO_4_ with those obtained for K_3_PO_4_ (Figure 3)—where an increase in the cation concentration did not lead to an increase in MDA reduction rates by the polysaccharide. This phenomenon can be explained by the anion effect detailed below.

Based on former reports which suggested that proteoglycans and proteins which are arrayed with carbohydrate polymers contribute to induction of polysaccharide antioxidant activities and therefore are probably involved in the polysccharide antioxidative protective activities [24,50,51,52,53,54], another possible explanation for the cation antioxidant effect can be postulated; a specific interaction of cations with the cell-wall proteins, which are part of the polysaccharides, probably through specific amino acid residues (most likely negatively charged) may occur. The interaction may cause conformational or structural changes, leading to better exposure of the antioxidant sites.

**Figure 4:**
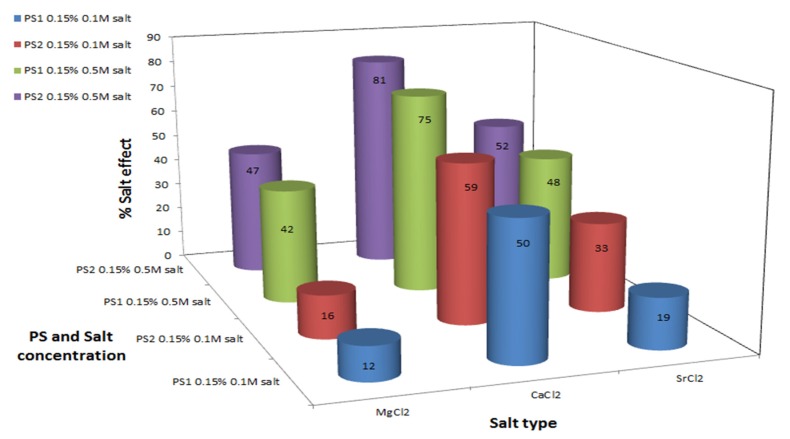
Polysaccharide antioxidant activity induction by divalent cation salts. C_MDA_ was measured by TBA test. Experimental setup included 0.5 g formula with 0.35 mM KO_2_, 0.15% *w*/*v* polysaccharide, and various divalent salts at two different concentrations, in a final volume of 10 mL. Values represent the mean of at least 3 repeats. Maximum standard deviation equals 8%.

Divalent cation type effect: Comparing the divalent cation treatments indicats that Ca^2+^ had the strongest influence on the reduction of MDA levels: 0.5M CaCl_2_ reduced MDA level by 75% and 81% in PS1 and PS2 formula-treatments, respectively (Figure 4). This finding is not surprising, since Ca^2+^ ions are known to be effective molecules that stabilize polysaccharides by creating a cross-linked network [55]; for example, Ca^2+^ ions are used for alginate gel formation, which is commonly explained by the egg-box model [56]. According to this model, the structural features of the glucoronic acid allow strong complexation with Ca^2+^ ions, which are embodied in cavities of stiff alginate chains like eggs in a cardboard egg box. Since PS1 and PS2 also contain anionic glucuronic acid blocks and sulfate groups, Ca^2+^ ions probably create a strong complexation with the anions, stabilizing the interactions between polysaccharide chains. The Ca^2+^ ions induced a significantly stronger enhancement in the antioxidant activity of both polysaccharides, in comparison to other divalent ions (Mg^2+^/Sr^2+^), see Figure 4. This enhancement is attributed to the conformational change of the polysaccharide which occurs due to the masking of its negative charges by the cation. It is likely that the cation’s charge density and radius play an important role in this sense—thus, we suggest that, of the three divalent cations tested, Ca^2+^ is best suited for rendering stability to the polysaccharide, due to the suitability of its charge density and radius.

The weakest antioxidant effect among the divalent ion treatments was observed in the low concentration MgCl_2_-polysaccharide treatments: 0.1 M MgCl_2_ reduced the MDA level by 12% and 16% in the PS1- and PS2-formula treatments respectively, compared to the same treatment without salt (Figure 4). This is also not suprising, since alginate hydrogels have been obtained with various divalent ions except for Mg^2+^ [57,58].

Anion type effect: It can be postulated that anion type also influence the polysaccharide antioxidant activity. A comparison between the effects of potassium and sodium sulfate salts (Na_2_SO_4_ and K_2_SO_4_), and between the potassium and sodium phosphate salts (Na_3_PO_4_ and K_3_PO_4_) point out that the type of anion does not only play a role in the induction of the polysaccharides’ antioxidant activity, but also differs between the two polysaccharides examined. For PS1 % salt effect values were much higher in the presence of SO_4_^2−^ in comparison to PO_4_^3−^ ions, the opposite effect was apparent in PS2 system, % salt effect values were higher in the presence of phosphate ions in comparison to sulfate ions, see Figure 2 and Figure 3.

The counter anion (e.g., PO_4_^3−^ in the presence of PS1-formula or SO_4_^−2^ in the presence of PS2-formula), can cause the salt and/or the polysacchride to aggregate. In the case of salt aggregation the actual concentration of the free cations which could bind to the PS is not the analytical concentration. In the case of polysaccharide aggregation, the concentration of available polysaccharide chains is reduced, leading to less interactions between the cations and the negatively charged residues within the polysaccharide chains. The different effect that the anions have on the two polysaccharides is probably due to their chemical structure difference.

Salt concentration effect: Salt concentration affects the antioxidant activity of both polysaccharides. High concentration of each salt (including NaCl/KCl/MgCl_2_/CaCl_2_/SrCl_2_), 0.5 M, was resulted in higher % salt effect, indicating that the polysaccharides act as better antioxidants in comparison to the low salt concentrations, 0.1 M (Figure 2, Figure 3 and Figure 4). The antioxidant activity is not linearly dose-dependent and differs in correspondence to the salt type, e.g., formula-polysaccharide treatments that contained 0.5 M KCl reduced the MDA levels up to two fold in comparison to treatments that contained 0.1 M KCl, see Figure 3. Increasing the salt concentration in the PS1-MgCl_2_/SrCl_2_ formula-treatments, Figure 4, also led to an increase in the antioxidant activity (up to three fold of MDA reduction). Noticeably, increasing the NaCl concentration had only a slight effect on the MDA levels in comparison to the other salts studied (Figure 2). The nonlinear effect of the salt concentration could be explained by the same explanation as was explained above in the anions effect. High concentration could cause to salt aggregation which reduced the actual free cation concentration in the solution. As a result less cation-polysaccharide interactions could be formed.

## 3. Experimental Section

### 3.1. Materials

All solutions were prepared from analytical-grade chemicals and double distilled water (DDW) that was passed through a Millipore setup at a final resistivity that was above 10 MΩ/cm.

All reagents and solvents were of analytical grade. Trichloroacetic acid (TCA), thiobarbituric acid (TBA), and butylated hydroxyl toluene (BHT), KO_2_, KCl, NaCl, K_2_SO_4_, Na_2_SO_4_, K_3_PO_4_, Na_3_PO_4_, MgCl_2_, CaCl_2_, SrCl_2_ were purchased from Sigma-Aldrich (Rechovot, Israel). Milk sample was a commercial newborn formula based on soy bean; its composition is given in Table S1 in the supplementary information.

### 3.2. Algae and Growth Conditions

*P.* sp. (UTEX 637) was obtained from Culture Collection of Texas University, Austin, Texas, USA. *P.* arg. (B111.79) was obtained from the culture collection of the University of Gӧttingen, Gӧttingen, Germany. Both species were cultivated in 250 mL Erlenmeyer flasks, each containing 100 mL of growth medium—*P.* sp. in artificial sea water (ASW) [59], and *P. arg.* in freshwater [45]. Algae were grown under shaking speed of 100 rpm; temperature of (25 ± 3) °C; illumination was supplied continuously from above with fluorescent cool-white lamps at a photon flux density of 90 µmol photons m^−2^·s^−1^; aeration was provided through bubbling air containing 2%–3% CO_2_ into the shaker. All cultures were inoculated with 48-h-old cells. Initial cell concentration was adjusted to 2 × 10^6^ cells/mL.

### 3.3. Isolation and Quantitative Analysis of Soluble-Sulfated Polysaccharide Fraction

Cultures at stationary phase of growth were centrifuged (3000 *g*, 10 min, 4 °C) and the supernatants containing the dissolved polysaccharides were dialyzed (MW cutoff 8000 Da) against DDW at 4 °C until the conductivity of the water reached 300 µs × cm^−1^. The dialyzed polysaccharide was then freeze-dried and resuspended in DDW to reach 1% *w*/*v*, comprising the PS stock. The polysaccharide concentration was determined by a known procedure [60], with D-galactose (Sigma G-0625, St. Louis, MO, USA) as the standard, in a concentration range of 0.1–1 µg/mL.

### 3.4. Sample Preparation

The sample preparation procedure included three steps: (1) Preparation of the PS-salt solution samples: The diluted PS solution (0.15% *w*/*v* in DDW) was incubated for 60 min with an appropriate concentration of one of the following salts: KCl, NaCl, K_2_SO_4_, Na_2_SO_4_, K_3_PO_4_, Na_3_PO_4_, MgCl_2_, CaCl_2_, SrCl_2_ at 0.1 M, or KCl, NaCl, MgCl_2_, CaCl_2_, SrCl_2_ at 0.5 M. The solubility of K_2_SO_4_, Na_2_SO_4_, K_3_PO_4_, Na_3_PO_4_ in aqueous solutions is lower than 0.5 M, therefore the only concentration used was 0.1 M; (2) Preparation of the PS-formula systems: The PS-salt solution samples were added to 0.5 g milk powder with 35 mM KO_2_, at a total volume of 10 mL; (3) Malondialdehyde (MDA) levels were determined according to the thiobarbitoric acid (TBA) method described below. Control experiments were performed separately in order to study the oxidation of the polysaccharides and the formula by KO_2_ (without salts) using the same procedure detailed above. To extend our knowledge regarding the dose-dependency of the polysaccharide effect on the milk powder without salt supplement, two polysaccharide concentrations (0.075/0.15% *w*/*v*) were studied for PS1 and PS2.

### 3.5. Production of Free Radicals

The free radicals were formed by addition of KO_2_ to the samples. As mentioned above O_2_^−^ is known as an oxidative agent [4,61].

The KO_2_ solution was prepared fresh for each experiment and added to the sample immediately after its preparation.

### 3.6. Malondialdehyde (MDA) Measurements

MDA levels were measured spectroscopically according to Fenaille *et al.* [62] with minor modifications. The assay is based on a reaction between oxidized lipids and TBA under acidic conditions to yield a pink chromogen with a maximum absorbance at 532 nm. After sample preparation an aliquot of the slurry (2 mL) was transferred to a 5-mL tube, to which the following were added: 1.6 mL DDW, 0.8 mL TBA 0.67%, 0.8 mL TCA 5% and 0.0128 g of BHT. The mixture was homogenized and centrifuged at 3500 rpm for 30 min. The supernatant was incubated in a 70 °C water bath for 60 min, and cooled to room temperature before measuring absorbance. The color produced by the chemical reaction was read at 532 nm in a Cary 100 Bio UV-Visible spectrophotometer, and the amount of MDA formed was determined by using the molar extinction coefficient ε(532 nm) = 1.56 × 10^−5^ cm·nmol^−1^ [62].

### 3.7. Calculation of % Salt Effect

The induction levels of the PS antioxidant activity under different salt environments was calculated according to the following formula: % salt effect = (A_0_ − A)/A_0_ × 100, where A_0_ is the absorbance of the control sample without salt, and A is the absorbance of the test sample with the salt.

### 3.8. Statistical Analyses

All data were expressed as means ± standard deviation (SD) of at least three replications, and the ANOVA test was used for statistical analysis. Each experiment was repeated at least three times.

## 4. Conclusions

Red microalgae polysaccharides hold great potential to be used as agents in various products for human benefit, for example, as natural antioxidants in food or cosmetic products. However, there are very few reports on sulfated red micoalgae polysaccharide antioxidant bioactivities and there are still many open questions to be answered regarding the mechanisms through which the polysaccharide copes with the ROS. The results of this study show that both red microalgae soluble-sulfated polysaccharide fractions (PS1 and PS2) exhibit significant radical scavenging abilities in soy bean formula in the presence of KO_2_.

It was demonstrated that salt composition (cation and anion type) and concentration affect the antioxidant activity of these red microalgal polysaccharides. Thus we can postulate that the salt ions intefere with the polysaccharide chain interactions by changing the intermolecular bonds and causing changes in the three-dimensional structure of the polysaccharide, leading to a newly formed structure that better exposes the antiooxidant sites. Among the cations which were tested, Ca^2+^ had the strongest enhancement effect on antioxidant activities of both polysaccharides. Since proteins are also part of the cell-wall complex polysaccharides [26,38] they can be involved in the newly-formed three-dimensional structure. It seems, however, that the activity of the polysaccharides does not result from one single component but from synergism of its oligomeric components. Due to the polysaccharide complexity and the lack of knowledge regarding its structure, the mechanism of the salt effect cannot be explained in depth.

Here we report for the first time the potential of the polysaccharides to act as an antioxidant ingredient in infant food formula, which can be used as a model for food products, as well as for other disciplines. We further demonstrate the enhancing effect of salts on this antioxidant activity and propose a hypothesis which can explain the differential effect of the various cations and anions on the two polysaccharides. The physicochemical properties which enable the polysaccharides to be used as a stabilizer or thickener, the relative ease in which they can be produced by controlling algal growth conditions and their uniqueness as a natural material which is dissolved in aqueous systems, reinforce their potential to be used as antioxidants in industrial systems. Our findings have a contribution in furthering knowledge on the polysaccharides’ antioxidant behavior under different environmental conditions.

## Supplementary Files

Supplementary File 1
