# Supplementary material for: Salt Effect on the Antioxidant Activity of Red Microalgal Sulfated Polysaccharides in Soy-Bean Formula
Source: Mar Drugs. 2015 Oct 20;13(10):6425–39. doi: 10.3390/md13106425 (PMC4626698; doi:10.3390/md13106425)
Supplement: Supplementary File 1 [file marinedrugs-13-06425-s001.docx]

**Supplementary Information**

**Table S1.** Formula composition of soy bean vegan milk.

| **Ingredients in 100g Powder** |  |
| --- | --- |
| Protein (g) | 16 |
| Carbohydrates (g) | 53 |
| Total fats g | 25 |
| Saturated fats (g) | 11 |
| Trans Fat (g) | <0.5 |
| Unsaturated fats (g) | 18.4 |
| Linoleic acid (mg) | 4580 |
| α-Linolenic acid (mg) | 420 |
| Sodium (mg) | 235 |
| Vitamin D (mcg) | 8.6 |
| Vitamin E (mg) | 13 |
| Vitamin C (mg) | 105 |
| Vitamin A (mcg) | 643 |
| Vitamin k (mcg) | 65 |
| Vitamin B1 (mcg) | 0.51 |
| Vitamin B2 (mcg) | 0.77 |
| Iron (mg) | 9.5 |
| Copper (mcg) | 378 |
